# A Pilot Study of a Novel Dietary Intervention Targeting Ultra‐Processed Food Intake

**DOI:** 10.1002/osp4.70029

**Published:** 2024-12-08

**Authors:** Charlotte J. Hagerman, Asher E. Hong, Emma Jennings, Meghan L. Butryn

**Affiliations:** ^1^ Center for Weight, Eating, and Lifestyle Science Drexel University Philadelphia Pennsylvania USA; ^2^ Department of Psychological and Brain Sciences Drexel University Philadelphia Pennsylvania USA

**Keywords:** behavioral intervention, dietary quality, obesity, ultra‐processed foods, weight loss

## Abstract

**Background:**

Ultra‐processed foods (UPFs) are harmful to health but ubiquitous in the modern food environment, comprising almost 60% of the average American diet. This study assessed the feasibility, acceptability, and preliminary efficacy of a novel behavioral intervention designed to reduce UPF intake.

**Methods:**

Fourteen adults participated in an 8‐week pilot intervention, which consisted of weekly group sessions, individual meal planning sessions, and financial support. Dietary intake was assessed using three Automated Self‐Administered 24‐h Dietary Recalls (ASA24) at both baseline and post‐treatment.

**Results:**

The intervention was highly feasible and acceptable. Qualitative data demonstrated that participants were enthusiastic about the benefits of reducing UPF intake and found the intervention highly valuable. Participants reduced average daily calories from UPF by 48.9%, number of UPFs consumed by almost half, total daily calorie intake by 612 calories/day, sodium consumption by 37% and sugar consumption by 50%. There were no significant changes in fruit or vegetable intake. Participants lost an average of 3.5 kg (SD = 3.0 kg).

**Conclusion:**

This pilot data suggests that behavioral interventions to reduce UPF intake will be well‐received and are capable of success despite the barriers of the United States food environment. Future research should prioritize behavioral interventions targeting UPF consumption alongside policy changes.

## 1 Introduction

Almost 60% of the average American diet is comprised of ultra‐processed foods (UPFs) [1]. UPFs are industrial formulations that consist of no or minimal whole foods and are produced with substances extracted from foods or synthesized in laboratories, such as dyes, flavorings, and preservatives, using industrial techniques that could not be recreated in the home, such as extrusion or molding [2]. Common examples of UPFs include breads, frozen meals, candies, sodas, cakes, cookies, salty snacks, and breakfast cereals [1]. UPFs were deliberately designed by food companies to be hyper‐palatable, low‐cost, convenient (e.g., shelf‐stable, ready‐to‐eat), and thus, highly profitable [2].

In the past decade, a large body of research has shown that UPFs are harmful to health. Intake of these foods is associated with obesity, type 2 diabetes, cardiometabolic diseases, depression, irritable bowel syndrome, cancers, and mortality [3, 4, 5, 6, 7]. In fact, many researchers believe that the primary reason obesity and weight‐related diseases in the United States have doubled since the 1970s is due to the increase in UPFs during this time [8, 9]. The effects of UPF on excess weight gain and other poor health outcomes have been attributed to their high caloric density and their poor macronutrient profile. Recent studies also provide strong evidence that food processing itself is harmful [10, 11, 12]. Although the exact pathways are unknown, the primary hypotheses for the detrimental effects of processing include food additives (e.g., colors, emulsifiers, preservatives), substances that arise during processing, and contaminants from food packaging, all of which may negatively impact absorption kinetics, satiety, glycemic response, and the gut microbiota composition and function [13, 14]. UPFs are more easily ingested than less processed food, meaning that calories can be consumed more rapidly [15, 16].

Despite the abundance of behavioral interventions to improve eating behavior in the United States, almost none have directly targeted UPF reduction [17, 18]. To date, the research team is aware of only one study, which was conducted with a small sample of food pantry clients (*N* = 43), that tested an intervention with the primary goal of reducing UPF intake [19]. The intervention provided food boxes along with education and social support through coaching calls. Indicators of dietary quality increased and calorie intake decreased, but the intervention did not report changes in overall UPF intake [19].

Outside the United States, a small collection of behavioral interventions to reduce UPF intake has been tested in Brazil, where research on UPFs originated. Brazilian interventions have shown mixed success. Two programs with pregnant women achieved small reductions in the percent of calories coming from UPFs (i.e., 4%–10% reduction in total calories coming from UPFs) [20, 21], whereas a larger study for pregnant women (*N* = 350), with less contact time, found no reduction in UPF intake [22]. One 6‐month intervention for adolescents (*N* = 62) led to reductions only in specific types of UPFs (soft drinks, sandwich cookies, and instant noodles) [23]. Some high intensity interventions (i.e., weekly group or individual sessions) have shown more impressive outcomes, for example, a 37.5% reduction in calories from UPF in a study with adolescents (*N* = 42) [24], and a 33%–46% reduction in a program for adults with metabolic syndrome (*N* = 70) [25].

Reducing UPF intake is expected to be extremely challenging, especially in the United States. Although Brazilian interventions have shown some success, Brazilians eat approximately *half* the amount of UPFs that Americans do, with only 20%–29.6% of daily calories coming from UPFs, compared to almost 60% in the United States [3]. The powerful UPF industry has ensured that UPFs are deeply ingrained in United States food culture, raising concerns about the practicality of behavioral interventions that target these foods [26]. UPFs comprise 58%–65% of the United States food supply and 73% of the food sold in grocery stores [27, 28]. A diet of mostly UPFs requires little planning, time, or culinary skill, which is well‐suited to the fast‐paced American food culture, in which culinary skills are on the decline [29]. Ultra‐processed meals and snacks are ready‐to‐eat or able to be prepared almost instantly with minimal skill (e.g., freezer meals) [2]. Further, UPFs are more affordable than minimally or unprocessed alternatives [30]. On average, UPFs cost $0.55 per 100 kilocalories, whereas unprocessed food costs $1.45 per 100 kilocalories in the United States [31]. The low cost of UPFs is attractive for most Americans, and necessary for others.

In addition to being deeply entrenched in American food culture, UPFs were deliberately designed by the food industry to be hyper‐palatable. Industrial formulations of UPFs include artificially inflated levels of saturated fats, refined carbohydrates (e.g., added sugars), sodium, and other additives (e.g., artificial sweeteners) that make them highly rewarding and difficult to resist [32, 33], providing a reward similar to drugs like cocaine [34]. The human brain, developed for regulating the intake of naturally‐occurring foods, is ill‐equipped to handle this level of reward [33, 35]. The reinforcing properties of UPFs disrupt the brain's reward system, such that the hedonic desire for food overrides satiety and fullness cues, promoting overconsumption [36, 37]. Similar to other addictive substances, consuming UPFs leads to increased tolerance, requiring greater consumption to achieve the same reward [36]. There is strong evidence that many people meet thresholds for conventional definitions of UPF addiction and experience adverse outcomes that are emblematic of drug addiction (e.g., symptoms of withdrawal) [32, 33].

Because UPFs are so challenging to reduce, it is unclear how an intervention to reduce UPF intake will be received in the United States. The present research team developed an intervention designed to address the many challenges of the United States food environment and culture. This intervention had five core components. First, it provided **education** on identifying UPFs and their harmful health effects. Participants also learned of the nefarious actions of the UPF industry to engineer and market these foods for profit. Second, participants received **meal planning sessions** that provided hands‐on support and knowledge for preparing more time‐intensive and minimally processed recipes. Third, they received **financial support** to experiment with purchasing whole or minimally processed foods at lower risk, and education about purchasing non‐UPFs on a budget. Fourth, to address the discomfort (e.g., cravings, withdrawal, feelings of deprivation) of reducing these highly palatable foods, participants learned **acceptance‐based strategies**, based on principles of acceptance and commitment therapy (ACT). In analog studies, ACT strategies have been shown to help people resist temptation to eat hyperpalatable food [38, 39], and adding these strategies to standard behavioral weight loss treatment has been shown to improve outcomes [40, 41]. In the current study, participants were taught ACT principles to mindfully accept feelings of discomfort, allowing them to make eating decisions determined by their long‐term values rather than momentary urges [42]. Finally, to limit participants' exposure to UPF, the intervention helped participants modify their **home food environment** with the help of another adult who lives with them. Some programs have targeted the home food environment for dietary change outside the context of UPFs and have yielded success [43, 44, 45]. Thus, modifying the home food environment and involving household members is expected to be an especially powerful strategy to reduce UPF intake.

The current study evaluated the feasibility, acceptability, and preliminary efficacy of this novel intervention.

*Aim 1* was to explore the feasibility and acceptability of a behavioral intervention to reduce UPF intake in the United States. Participants' satisfaction with the intervention, individual components of the intervention, and their experiences reducing UPF intake were also reported.

*Aim 2* was to test the preliminary efficacy of the intervention in reducing UPF intake, improving indicators of dietary quality (i.e., sodium, added sugar, saturated fat, fruit, and vegetable intake), and achieving weight loss.

## 2 Materials and Methods

### 2.1 Participants

Participants were recruited from waitlists of those seeking behavioral treatment to improve their eating behavior at the Center for Weight, Eating, and Lifestyle Science (WELL) of Drexel University. To be eligible, participants were required to be between ages 18–75, to have a desire to reduce their UPF intake, and to report consuming at least two UPF items per day with at least four distinct types of UPF items in the past week. They also could not have a medical condition that might pose a risk to their participation. Finally, participants needed to live with another adult willing to serve in a support role during the intervention and attend two group sessions with them.

The sample (*N* = 14) included 85.7% women (*M* age = 49.8 years (SD = 15.2 years); 50% White, 42.9% Black, and 1 participant who did not specify). Although it was not a requirement to participate, all participants were overweight or had obesity (*M* Body Mass Index = 42.5 kg/m^2^, SD = 11.7 kg/m^2^), and all participants indicated that they wanted to lose weight strongly (14.3%) or very strongly (85.7%). Household members (42.9% women, *M* age = 51.3 years, SD = 14.17 years) were mostly spouses or romantic partners (85.7%), along with one parent and one adult child.

### 2.2 Intervention

#### 2.2.1 Group Sessions

For 8 weeks, all participants attended weekly group sessions with a trained lifestyle modification coach. Group sessions involved didactic components, discussion, and activities. Please see Table 1 for the schedule of treatment and content of sessions.

**TABLE 1:** Intervention schedule and content.

Week	Group session theme	Content covered	Materials employed	Household member attended group	Individual meal planning meeting
1	Introductions	Introduction to program, UPF education, self‐monitoring, household support	Didactic (slides), group discussion	X	X
2	Substitutions and cravings	UPF substitutions, handling cravings, addiction and willingness education, urge surfing introduction	Didactic (slides), group discussion		
3	Meal planning	Introduction to planning meals, grocery shopping, budgeting, planning snacks	Didactic (slides), group discussion, activity		X
4	Household support	Stimulus control, support versus criticism, household member involvement	Group discussion, activity	X	
5	Substitutions and cravings (continued)	Handling cravings, substitutions, environmental changes for reducing UPFs	Didactic (slides), group discussion		X
6	Mindfulness and willingness	Mindful decision‐making, knowledge, mindfulness, willingness	Didactic (slides), group discussion		
7	Check‐in and review	Self‐monitoring check‐in, revisiting stimulus control	Group discussion, activity		X
8	End of program	Program reflection, ASA24 food recall instructions, focus group	Focus group		

During these sessions, participants received education about identifying UPFs and their harmful effects. They also learned the intentional harms of the food industry in creating these foods. During group sessions, participants also learned **acceptance‐based strategies** for coping with the cravings and withdrawal of reducing ultra‐processed food. Techniques included values clarification (i.e., identification of long‐term values and how healthy behavior supports these values), experiential acceptance (i.e., willingness to tolerate uncomfortable experiences in service of these values), and mindful decision‐making (i.e., the practice of choosing behaviors in line with personal values rather than immediate desires) [42]. Group sessions also incorporated **household** **support**, stressing the importance of the household environment for reducing UPF intake. Each index participant was asked to bring their designated adult household member to two specified group sessions (Sessions 1 and 4), during which the coach addressed the role of the entire household in improving the home food environment and taught effective communication strategies.

#### 2.2.2 Meal Planning Coaching Sessions

Index participants also completed four biweekly, 30‐min meal planning sessions with a coach during which they identified meals and snacks with no or minimal UPFs to prepare. All participants were asked to track their daily food intake using a widely available free app, and food records were regularly reviewed by the meal planning coach for feedback. Participants interested in weight loss (which in this case, was all participants) were encouraged to consider caloric intake when meal planning. They were taught that reducing UPF was an excellent way to reduce calorie intake, but that they should be mindful of the calorie content of the non‐UPF swaps that they chose as well.

#### 2.2.3 Financial Support

To support the experimentation with new, non‐UPF foods and recipes with minimal financial risk, participants received a $100 gift card to a grocery store of their choice. Participants were encouraged to use this gift card to purchase minimally processed and whole foods.

### 2.3 Measures

All measures of dietary intake were assessed using the Automated Self‐Administered 24‐h Dietary Recall (ASA24), a well‐validated, web‐based measure of comprehensive dietary intake developed by the National Institutes of Health provided at no cost [46]. At baseline and post‐treatment, participants completed three recalls reporting all foods and beverages they consumed during the past 24 h (two weekdays, one weekend day). Participants were told to complete the recalls at the soonest available weekday and weekend days; however, if they forgot or had technical difficulties, they were instructed to complete it at the next soonest weekday or weekend day (depending on what they had missed). Most participants completed the recalls during a 4‐day span (85.7% at baseline and 84.6% at post‐treatment), and all completed them within 6 days. At baseline, an assessor walked the participants through the assessment tool and completed the first 24‐h assessment with the participant. All subsequent recalls were self‐administered.

UPF intake (primary outcome) was measured as the average daily calories from UPFs, average percent of daily calories from UPFs and unique number of UPF items per day. Although the ASA24 does not provide data on food processing level, a member of the study team classified each recorded food as UPF or not, according to NOVA classification definitions. To mitigate bias, the assessor was blind to the study timepoint. Ambiguous food items (15.3% of the total items classified by the original assessor) were discussed with a collaborator with expertise in UPF (who was also blind to condition), and consensus was reached. To aid in classification, reviewers used context clues provided by the ASA24, including nutrient content (specifically added sugar, sodium, and fat) as well as where the food was sourced from (e.g., supermarket, produce stand, fast food, etc.). The ASA24 automatically generates data on dietary components, including teaspoons of added sugar, milligrams of sodium, grams of saturated fat, cups of fruit, cups of vegetables, and average daily calorie intake. All of these measures were also examined as outcomes.

Participants also self‐reported their weight in pounds at baseline and post‐treatment. They were given instructions to weigh themselves first thing in the morning. Finally, all participants attended a final focus group, moderated by a researcher who was not involved in delivering the intervention and was thus unknown to participants (CH). During the focus group, they shared their qualitative feedback and anonymously provided responses to survey questions about their experiences. Participants reported their perceptions of the extent to which they and their household members had reduced their UPF intake on a scale from 1 (*did not change*) to three (*eating a lot fewer UPFs than before*). They rated their overall satisfaction with the program on a scale from 1 (*not at all satisfied*) to 3 (*very satisfied*) and the perceived helpfulness of different components of the intervention on a scale from 1 (*not at all helpful*) to 3 (*very helpful*). Through open‐ended questions, participants were asked to elaborate on their responses. Participants were also asked to discuss the benefits and barriers of reducing UPF intake they experienced throughout the program.

### 2.4 Statistical Analysis

Descriptive statistics reported responses to quantitative focus group questions. Exemplary quotes were pulled from qualitative feedback. Paired sample t‐tests were used to examine differences from baseline to post‐treatment; *t*‐values, *p*‐values, and means are reported. The sole participant who was lost to follow‐up was not included in the analysis.

## 3 Results

### 3.1 Feasibility and Acceptability

The intervention was feasible. Of the 40 participants who were screened for eligibility, 92.5% expressed interest in the study after hearing the description. However, 16 participants were ineligible (4 were older than 75, 4 had a medical condition that excluded them from participating (e.g., kidney disease), 4 did not consume enough UPFs, 2 did not have household members, 1 had a history of bariatric surgery, and 1 had a scheduling conflict). The remaining 24 participants were scheduled for a baseline assessment, but 3 did not show up, and 1 was excluded because their household member enrolled (this person opted to participate as a household member rather than as an index participant). An additional 2 participants were excluded after baseline because they had scheduling conflicts, and 4 did not complete the necessary enrollment tasks (3 ASA24 recalls).

The remaining 14 participants enrolled, and only one dropped out of the study (lost contact after session 1). Of the remaining 13 participants, 53.8% attended all 8 sessions, 38.5% attended 7 sessions, and 7.6% attended 6 sessions. Household member attendance was also successful; all household members attended both planned sessions except for one, who attended only one of the sessions.

The intervention was highly acceptable. In the final focus group, 84.6% of participants reported being very satisfied, and 15.1% reported being satisfied with the intervention. All participants perceived that they were consuming fewer UPFs; 69% believed they were eating much less and 30.1% believed they were eating somewhat less.

### 3.2 Changes in Dietary Intake and Weight

See Table 2 for means of all outcomes at baseline and 8 weeks. Participants reduced their average daily calories from UPFs by almost half (48.9%; *t*(12) = 4.21, *p* = 0.001) and the average daily number of UPF items they consumed by almost half (*t*(12) = 4.18, *p* = 0.001). The total percentage of daily calories from UPF also significantly declined by 23.7% (*t*(12) = 4.04, *p* = 0.002).

**TABLE 2:** Average daily intake measures of dietary quality at baseline and at the end of treatment (8 weeks).

	Daily mean at baseline (SD)	Daily mean at post‐treatment (SD)	*p*‐value
Calories from UPF (Kcal)	1944.3 (924.4)	992.7 (722.1)	*p* = 0.001**
Percent of total calories from UPF (Kcal, percent)	74.4 (14.4)	50.5 (21.9)	*p* = 0.002**
Number of UPF items	11.5 (4.02)	6.2 (3.7)	*p* = 0.001**
Saturated fat (g)	38.0 (16.7)	23.9 (16.7)	*p* = 0.006**
Added sugar (tsp)	23.1 (16.3)	11.1 (13.5)	*p* = 0.004**
Sodium (mg)	4366.1 (1844.3)	3124.6 (1459.3)	*p* = 0.014*
Fruit intake (cups)	0.7 (0.9)	1.1 (1.0)	*p* = 0.167
Vegetable intake (cups)	2.2 (1.6)	1.9 (1.3)	*p* = 0.280
Total calorie intake (Kcal)	2561.5 (1053.2)	1949.0 (1251.7)	*p* = 0.015*

*p < 0.05; **p < 0.01.

Saturated fat consumption decreased by approximately 37% (*t*(12) = 3.29, *p* = 0.006) and added sugar consumption was reduced by approximately 50% (*t*(12) = 3.49, *p* = 0.004). Participants also consumed approximately 28% less sodium at posttreatment (*t*(12) = 2.89, *p* = 0.014). Total daily calorie intake decreased by approximately 612 calories/day (*t*(12) = 2.83, *p* = 0.015). However, there were no significant changes in fruit intake (*t*(12) = ‐1.47, *p* = 0.167) or vegetable intake (*t*(12) = 0.60, *p* = 0.280).

Participants also reported losing an average of 3.5 kg (SD = 3.0 kg) during the 8‐week intervention (*M* baseline weight = 118.8 kg (SD = 33.4 kg); *M* post‐treatment weight = 115.3 kg (SD = 33.0 kg); *t*(13) = 4.21, *p* < 0.001).

### 3.3 Qualitative Feedback

Important themes from the final focus groups and exemplar quotes are noted below.

#### 3.3.1 Benefits of Reducing UPF Intake

When asked about the benefits of reducing their UPF intake, participants were enthusiastic in reporting the physical health benefits that they had experienced, including weight loss, physical comfort, and improved appearance.

I was in a weight loss journey already; I was in a stall. So eliminating UPFs was very helpful…I'm like, okay, I can continue this because now, the scale’s moving.

(Participant 9)

Much, much less swelling in my hands and in my ankles.

(Participant 4)

They also reported mental health benefits and improved mood.

I have way more energy…my attitude feels better.

(Participant 5)

I'm not as angry as I was. And I know that it has to have something to do with the study.

(Participant 12)

Several participants also reported that their cravings for UPFs declined throughout the study.

I have noticed my cravings for, like, snacks and sweets has gone down, and like I don't even want it anymore, which is good cause I was having really bad craving issues. And it was just interesting to know, like…these foods are super addictive.

(Participant 11)

I cut out soda. Now, it's like, I don't really want it. I don't want [it] that sweet.

(Participant 5)

#### 3.3.2 Barriers to Reducing UPF Intake

When asked about the major barriers to reducing UPF intake, several participants reported that special occasions and eating outside the home were the biggest barriers.

It's Christmas time, you know, you associate certain things with certain activities. And so I need to change that mindset because I'm good at home. It’s holiday cookie time, and that's part of it.

(Participant 4)

The biggest challenge I’ve been finding is when I'm traveling, when I'm eating away from home.

(Participant 3)

As expected, participants also noted that preparing meals without UPFs required more time and preparation.

For me, the biggest hurdle was really allocating enough time to do it, to make dishes that I know the family likes.

(Participant 2)

I find that when I don't prep, that’s when I have the most issues.

(Participant 10)

One participant who wanted to lose weight noted that many low‐calorie options are ultra‐processed, which presented a challenge.

When you're trying to lose weight—a lot of the lower calorie foods are all UPFs, things that you substitute in place of already.

(Participant 13)

#### 3.3.3 Reactions to Intervention

When asked about their responses to the overall intervention, participants reported that the comprehensive education on UPFs was highly valuable. Even those who thought themselves to be knowledgeable about UPFs before the study were able to build their existing knowledge.

I thought a lot of the things that I used to eat were okay. Then I started reading and seeing all the detailed little stuff in the packaged foods and realized they aren't, even if they're labelled [as healthy]. Knowledge has helped me.

(Participant 14)

One of my go‐tos was the crystal light packets. I didn't realize that those packets, even though they're marketed as low calorie, better for you, no sugar, etc., have so many chemicals in it. I've completely cut those out.

(Participant 9)

All participants reported that the **acceptance‐based strategies** for handling cravings for UPFs were helpful (84.6% said they were very helpful, 15.3% said somewhat helpful).

[The program] helped me to not only recognize UPFs but understand why we were craving those things and learn how to get beyond that. Urge‐surfing and [other acceptance‐based strategies] certainly were a very important part of [the program].

(Participant 4)

Participants had highly positive feedback for the biweekly **meal planning coaching sessions**; all participants found them helpful (84.6% very helpful, 15.3% somewhat helpful). Participants reported that the meal planning coach pushed them to be more adventurous with their food choices and identify meals.

[The coach] gave us all kinds of recipe ideas that my wife and I really liked, and it just turned us on to all these foods we didn't know before, and ways to prepare foods and make them taste excellent.

(Participant 5)

**Household support** was perceived as helpful for most, but not all, participants (46.2% said very helpful; 30.7% said somewhat helpful; 15.4% said not at all helpful). Approximately 61.6% of participants reported that their household members were eating somewhat or a lot less UPFs than they used to. Some participants reported that their household members were not agreeable to making changes themselves but were supportive of the participant doing so. These participants appreciated that the intervention did not require household members to change their consumption patterns with them, and they were able to benefit from the program whether their household members did or did not.

[My partner] was definitely supportive but didn't change anything himself, but I liked that [the program] acknowledged that and didn't require your household number to change their diet either.

(Participant 11)

I like the fact that they were included. I think someone had said not too long ago that it would have been nice to have them on board the whole time.

(Participant 9)

Others reported that their household members did not contribute much to their experience.

[I received the] bare minimum from him.

(Participant 14)

**Financial support** was perceived as helpful for almost all participants (84.6% said very helpful; 7.7% said somewhat helpful; 7.7% said not at all helpful). Most participants expressed that the financial support allowed them to experiment with new minimally processed and whole foods without the guilt of disliking the food or the fear of wasting money. Participants described that financial support took the burden and risk away from trying new foods.

Right before I had gotten my [financial support], [the meal planning coach] had given us a bunch of suggestions on different meals to try and I didn't have the money to go buy it. So, when that came in, I took that and bought [ingredients] for the meals that she had suggested.

(Participant 6)

I don't buy vegetables a lot because I don't like them and if I do want to try something, it's like, ‘Oh, I don't like it. I'm [going to] be wasting money. So, I'll just try this other [food] that I know about.’ So having the gift card is really helpful, because it was like, ‘Oh, I can like, go buy this thing. And it's okay if I don't like it, cause like it was free money, anyway.’

(Participant 11)

## 4 Discussion

The current study was one of the first to test a behavioral intervention to reduce UPF intake in the United States. The intervention demonstrated excellent feasibility and acceptability in this sample of participants who were highly motivated to change their eating behavior, with high attendance and ratings of satisfaction with the overall program. Qualitative feedback through focus groups at the end of treatment demonstrated that the intervention was perceived positively. Even with the challenges posed by the food environment in the United States, participants were enthusiastic about the intervention, amenable to reducing their UPF intake, and able to make changes consistent with program recommendations.

Large reductions in UPF intake were observed. The participants’ overall percentage of daily calories from UPFs declined by approximately 24%. This percentage actually underestimates the effect of intervention, as the number is biased by the marked decline in *overall* calorie intake from baseline to posttreatment (discussed below). When absolute numbers were examined, participants consumed approximately half the amount of UPFs at posttreatment as they did at baseline. Further, the intervention led to significant improvements in several indicators of dietary quality, including saturated fat, added sugar, and sodium consumption. Of note, there was no significant difference in fruits or vegetables consumed from pre to post‐treatment, suggesting that reducing UPF intake is not mutually exclusive with increasing consumption of fruits and vegetables. Interventions that aim to improve diet quality more globally may need to more strongly encourage the increase in these healthful foods while reducing UPFs.

In addition to these promising reductions in UPF intake and improvements in dietary quality, no participants reported barriers to reducing UPF intake that were insurmountable during the short‐ or long‐term. It is possible that the intervention successfully addressed the major barriers to reducing UPF intake, such as financial burden and inconvenience. Participants also reported noticeable physical benefits, such as better skin and reduced swelling in the limbs, even during such a limited study period. They also reported better mood and energy, which is consistent with past literature demonstrating that UPF intake is associated with poor mental health [47]. The salience of these benefits is promising, as these benefits are likely to perpetuate participants' motivation to change.

All participants reported that the meal planning sessions were helpful. Given the time investment, knowledge, and burden of preparing less processed meals, providing one‐on‐one support may be critical for helping participants make changes. The intervention content on acceptance‐based strategies was also well‐received, suggesting that participants can learn these skills and apply them to their eating behavior with relatively little instruction. The perceived helpfulness of household member involvement was generally positive but more mixed, particularly among participants whose household members were less supportive or willing to change themselves. In a larger sample, there are likely to be more instances of unsupportive or begrudging household members. However, many participants reported that their household members had reduced their own UPF intake, suggesting that involving household members may increase the reach of the intervention. As expected, financial support proved crucial for some participants but less helpful to others. Future research should test the isolated effectiveness of these intervention strategies and examine potential trait‐level moderators.

UPFs tend to be calorically dense, nutrient poor, and ultra‐palatable, leading to excess energy intake [31]. In the current study, participants consumed striking 600 fewer average daily calories by reducing UPFs. They also lost an average of almost 3.5 kg during the 8‐week intervention. These findings are consistent with a recent inpatient study by Hall and colleagues, in which participants were randomized to an ad‐libitum diet of UPF‐only or unprocessed‐only food for 2 weeks, followed by an alternate diet for 2 weeks [16]. Daily energy intake was an average of 500 calories higher during the UPF diet compared with the unprocessed diet, and participants lost an average 0.9 kg during the unprocessed diet period [16].

Because UPF intake declined by almost 1000 calories, yet overall intake declined by 600 calories, participants necessarily increased their consumption of other non‐UPF food swaps. Exploratory analyses found that, in addition to non‐significant increases in fruits and vegetables, there were no significant increases in nuts/seeds, eggs, unprocessed meat, or legumes during the study (*ps* > 0.05). Therefore, it is likely that participants increased their intake of many non‐UPF food groups, but did not increase their intake of any single group enough to reach significance.

The significant weight loss in this study is noteworthy given the limited focus on weight loss counseling within the program. The rate of weight loss observed in the current study is comparable to that achieved by traditional behavioral weight loss programs, which aim for a rate of 0.5–1 kg per week [48, 49]. Nevertheless, these preliminary results suggest that reducing UPFs is a promising and highly acceptable way to reduce calorie intake while achieving other physical and mental health benefits.

The most notable limitation of this pilot study was its small sample size; results should be interpreted with caution and cannot be assumed to be generalizable. However, to measure weight, this study relied on self‐reports, which may be inaccurate [49], and particularly subject to social desirability bias at the end of treatment. Eating behavior may have also been subject to this social desirability bias. If so, the findings reported here may be inflated.

All participants had overweight/obesity and were highly motivated both to lose weight and to reduce their UPF intake, as evidenced by their willingness to complete rigorous screening tasks to be eligible for the study (e.g., three 24‐h food recalls). Therefore, the results may not generalize to populations with lower motivation to change their diet. Reducing UPF intake has health benefits that extend far beyond weight loss (e.g., reduced cancer risk, mental health benefits) [3]; however, it is unclear whether people with lower BMIs would have sufficient motivation to see benefit from the intervention. The intervention may also have been less effective for individuals with less opportunity for improvement in their diet, that is, lower baseline intake of UPFs. The current study's sample had a higher baseline intake of UPFs (74% of daily calories) compared to the general population (approximately 60% of daily calories). This discrepancy could be due to the fact that the sample was overweight/obese, which is associated with higher UPF intake [50]. However, the discrepancy could also be due to differences in the tools used to measure UPF intake. Although this study used the ASA24, previous estimates have used in‐person interviews, which was not feasible for this project [1]. Although the ASA24 provides a rich, item‐by‐item measure of food intake, which is more rigorous than food frequency questionnaires, the current study's system for classifying foods as UPFs was certainly imperfect. There is a pressing need to create and validate low‐burden tools specifically for measuring UPF intake, which will allow for the most accurate measurement and comparison across studies.

The intervention was also short‐term, and participants may struggle to maintain reductions in UPF intake for longer periods of time. Future studies should include a well‐powered, longer‐term randomized controlled trial designed to evaluate the efficacy of this intervention in targeting UPF intake. A dismantling study could also help disentangle the relative utility of the study's components (e.g. meal planning, financial support, group sessions).

Although participants reported the financial support provided by the study to be helpful, this component of the intervention may be more difficult to disseminate and scale, as stakeholders may not have the financial resources to do so. Finally, while this was a behavioral intervention, there is also a dire need for policy change. To reduce UPF intake on a broad scale, integrated approaches that combine individual‐level interventions with broader public health policies are needed.

## 5 Conclusions

This pilot data suggests that behavioral interventions to reduce UPF intake will be well‐received and are capable of success despite the many barriers of the United States food environment. The marked improvements in dietary quality and weight, paired with the physical and mental health benefits that participants reported, suggest that UPFs are a promising intervention target for improving overall health and wellbeing. The intervention incorporated multiple components designed to address the many challenges of reducing UPF intake, and all were perceived positively. Future research should prioritize the development, adaptation, and strengthening of behavioral interventions targeting UPF consumption, alongside the implementation of policy changes that foster environments more conducive to healthy eating.

## Author Contributions

**Charlotte J. Hagerman:** conceptualization, methodology, investigation, formal analysis, data curation, writing–original draft, review, and editing. **Asher E. Hong:** literature Search, data curation, visualization, writing–review & editing. **Emma Jennings:** literature Search, writing–review & editing. **Meghan L. Butryn:** conceptualization, supervision, writing–review & editing.

## Ethics Statement

This study was reviewed and approved by the Drexel University Institutional Review Board on June 20, 2023 (protocol #2305009939). All procedures were conducted in accordance with the 2013 revision of the Declaration of Helsinki. Informed written consent was obtained from all participants prior to their participation in the study.

## Conflicts of Interest

The authors declare no conflicts of interest.
